# Perennial Grass and Native Wildflowers: A Synergistic Approach to Habitat Management

**DOI:** 10.3390/insects8040104

**Published:** 2017-09-22

**Authors:** Shereen S. Xavier, Dawn M. Olson, Alisa W. Coffin, Timothy C. Strickland, Jason M. Schmidt

**Affiliations:** 1 Department of Entomology, University of Georgia, Tifton, GA 31793 USA; shereensxavier@gmail.com; 2 Crop Protection and Management Research Unit, USDA-ARS, Tifton, GA 31793, USA; dawn.olson@ars.usda.gov; 3 Southeast Watershed Research Laboratory, USDA-ARS, Tifton, GA 31793, USA; alisa.coffin@ars.usda.gov (A.W.C.); Tim.Strickland@ars.usda.gov (T.C.S.)

**Keywords:** agroecosystem design, bioenergy grass, conservation, floral provisioning, functional groups, habitat management, LTAR, natural enemies, landscape restoration, sustainable intensification

## Abstract

Marginal agricultural land provides opportunities to diversify landscapes by producing biomass for biofuel, and through floral provisioning that enhances arthropod-mediated ecosystem service delivery. We examined the effects of local spatial context (adjacent to woodland or agriculture) and irrigation (irrigation or no irrigation) on wildflower bloom and visitation by arthropods in a biofeedstocks-wildflower habitat buffer design. Twenty habitat buffer plots were established containing a subplot of Napier grass (*Pennisetum perpureum* Schumach) for biofeedstock, three commercial wildflower mix subplots, and a control subplot containing spontaneous weeds. Arthropods and flowers were visually observed in quadrats throughout the season. At the end of the season we measured soil nutrients and harvested Napier biomass. We found irrespective of buffer location or irrigation, pollinators were observed more frequently early in the season and on experimental plots with wildflowers than on weeds in the control plots. Natural enemies showed a tendency for being more common on plots adjacent to a wooded border, and were also more commonly observed early in the season. Herbivore visits were infrequent and not significantly influenced by experimental treatments. Napier grass yields were high and typical of first-year yields reported regionally, and were not affected by location context or irrigation. Our results suggest habitat management designs integrating bioenergy crop and floral resources provide marketable biomass and habitat for beneficial arthropods.

## 1. Introduction

Within intensely managed agricultural landscapes, arthropod functional group diversity has declined, resulting in a loss of ecosystem services they typically provide (reviewed in [1]). In recent decades, applied research has focused on mitigating ecosystem service losses via habitat management. As local and landscape factors interact, Landis [1] proposes ecosystem services loss due to landscape simplification can only be addressed by a concerted effort to fundamentally redesign agricultural landscapes.

Marginal lands provide an opportunity for the provisioning of biodiversity and crop protection. In this context, marginal lands are areas where cultivation is possible and may have once occurred, but where conservation benefits strongly favor removal of these lands from active crop production (e.g., reviewed by [2]). Incorporating biofuel crops with wildflower habitats on marginal land may provide an opportunity to develop landscape designs better suited to increase biodiversity and ecosystem services, while mitigating competition with production of food and fiber. Analyses of the Coastal Plain of Georgia, USA, identified over 300,000 hectares of marginal land characterized by cropland-forest edges near riparian buffers and potential grassed waterways within agricultural landscapes [2].

Habitat management through the addition of native flowering plants to cropping systems is gaining momentum as an option for growers to increase ecosystem service delivery. For example, maintaining flowering plants both within fields and along their edges successfully enhances two important arthropod-mediated regulating services in agroecosystems: biological control of annual crop pests by natural enemies [3,4,5,6,7,8,9] and pollination of annual crops [10,11]. Such regulating services increase crop yield, with an added benefit of reducing the need for costly and environmentally risky pesticide application. Natural enemies and pollinators require food resources for enhancement of longevity, fecundity, and foraging activity [9]. Therefore, continuous availability of floral resources in an agricultural landscape may be essential to promote healthy beneficial arthropods, thereby increasing their ability to deliver effective biological control and pollination services to annual crops [12].

Perennial bioenergy crops can also increase the biodiversity of multiple taxa and sustain a variety of ecosystem services, such as pest suppression, pollination, and erosion prevention [13]. As the Energy Independence Security Act (EISA) mandates an increase in biofuel production to 21 billion gallons by 2022 [14], the southeastern region of the USA has been identified as a major contributor due to high yields of warm season grasses [15]. Previous studies of perennial grasses performance in the Coastal Plain of Georgia indicate *Pennisetum purpureum* (Napier grass), *Miscanthus × giganteus* and *Panicum virgatum* (switchgrass) are equally suitable biofuel crops [2,15,16]. The use of perennial grasses for biomass production could provide an opportunity to ecologically intensify agricultural landscapes. The goal of ecological intensification is to make quantifiable direct or indirect contributions to agricultural production, while reducing environmental impacts, such as biodiversity loss [17]. For example, perennial grasslands support higher natural enemy abundance, diversity, and biomass compared to annual crops such as maize [18], and grasslands containing forb cover and flowering species support higher abundance of natural enemies and associated biocontrol services [19]. Agricultural designs combining perennial grasses and floral resources have the potential to synergistically increase provisioning of habitat for beneficial organisms while providing biofuel feedstocks as an additional grower commodity.

Habitat management program success is context dependent, where local and landscape factors influence the benefits of additional habitat to a cropping system [20,21]. Two local factors to be considered in habitat management are irrigation and soil nutrients, as some perennial grasses such as *Miscanthus × giganteus* and switchgrass suffer reduced biomass yields and biomass quality under limited water and soil nutrient levels [16,22,23]. To the best of our knowledge, effects of soil nutrient and pH levels have not been considered in wildflower strip establishment. Another contributing local factor is the type of habitat adjacent to crop fields (e.g., woodland or agriculture). Ingrao et al. [24] found natural enemy abundance and pest control were higher within woodland habitat and adjacent crop field edges compared to within crop field interiors. Therefore, we ask how do arthropods and wildflowers respond to woodland or agriculture location context and irrigation in early establishment of habitat buffer programs. Our specific objectives were to (1) quantify flower visits of arthropod natural enemies, pollinators and herbivores on several native floral mixes in buffers combining Napier grass and wildflowers on marginal land, (2) test buffer location (adjacent to woodland or agriculture) and irrigation effects (irrigation or no irrigation) on wildflower counts, counts of arthropods on flowers, and Napier yield, and (3) provide initial data on nutrient correlations to wildflower production and Napier biomass.

## 2. Materials and Methods

### 2.1. Study Sites

**Figure 1:**
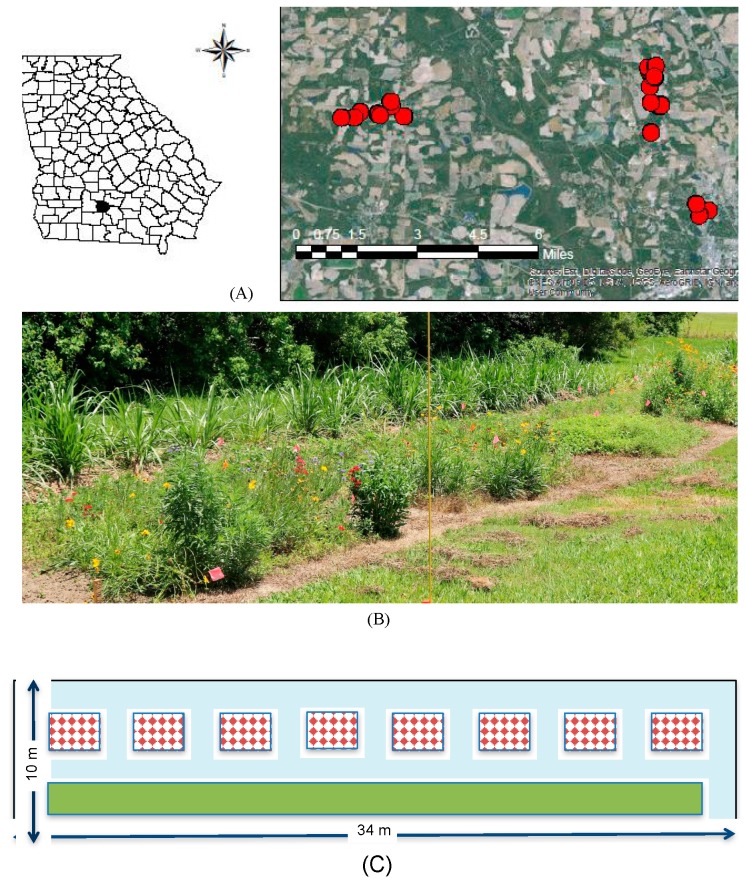
Buffer plots distributed across experimental farm stations at University of Georgia in Tift County, Georgia (**A**), photograph of experimental plot (**B**). Bottom panel (**C**) with plot design including eight randomized wildflower subplots (red checkered boxes) and one Napier grass subplot (green box), with vegetation free allies around all subplots (blue).

Twenty conservation buffer sites were selected on marginal land across University of Georgia experimental farms (Tifton, GA, USA, Tift County; Figure 1A). Buffer plots were assigned to a 2 × 2 design of local spatial context and irrigation. For local spatial context, ten plots were located adjacent to woodland (“T”) and ten in open areas between 1 and 30 m from agricultural fields (“A”). Half of these plots received irrigation or no irrigation. Irrigation treatments were irrigated (“I”) weekly from 5 April 2016–18 August 2016 at a moisture level of 2 cm based on a rain gauge. The other half of the plots received no additional irrigation (“N”). In late winter, plots were sprayed with Roundup^®^ (Monsanto, Melbourne, Australia) at 4.68 L/ha for weed suppression. A week later, a deep till rig (35.5 cm depth) was used to prepare the soil. A field cultivator was used to remove further weeds and smooth the soil for planting.

Each experimental buffer plot was 34 m × 10 m (340 m^2^), separated by distances of at least 150 m. Plots contained a 2 m × 30 m (60 m^2^) strip of Napier grass separated by a 2 m vegetation free alley on all sides (Figure 1B,C). Eight subplots, with final dimensions of 1.75 m × 2.6 m (~4.55 m^2^), were randomly assigned a wildflower treatment (Figure 1C). The area between wildflower subplots was sprayed with Roundup^®^ at 4.68 L/ha, and maintained free of vegetation.

To establish Napier grass (*Pennisetum purpureum* Schumach), ~9 cm (3.5 in) of the stems were cut from existing nearby plots near Tifton, GA, and grown in the greenhouse in pots with 3 parts potting soil (21:07:14 NPK) and 1 part sand, and watered as needed. Five grams of fertilizer (16:04:08 NPK) was applied monthly. The Napier grass was periodically cut to maintain height at 30.5 cm and was transplanted to the buffer plots on 5 April 2016. Napier grass was equally spaced ~91 cm apart in two alternating rows. Sixteen grams of fertilizer was applied at the base of each plant after planting.

The eight wildflower subplot planting treatments included (Figure 1B): two subplots, each with a unique flowering species; one subplot with a combination of the three species; three subplots with distinct commercial mixes; and one subplot containing spontaneous weed growth as the control. The wildflower subplots containing seeds of *Monarda fistulosa* and *Monarda citriodora* (Lamiaeae), and a combination of the two had poor germination/establishment so only data from the three commercial mixes and the control subplots were analyzed. The commercial seed mixes were specific to the southeastern USA (Southeast wildflower Seed Mix; see Table S1): Eden Brothers^®^ (Arden, NC, USA), High Country Gardens^®^ and American Meadows^®^, (Shelburne, VT, USA). The different wildflower mixtures are hereafter referred to as control (C), floral mix 1 (M1), floral mix 2 (M2) and floral mix 3 (M3), respectively. Following manufacturer instructions, the wildflower seeds (22.67 g) were mixed with 5 parts sand and sown in the plots by hand broadcasting on 7 December 2015.

### 2.2. Arthropod and Vegetation Sampling

Vegetation and arthropod sampling took place simultaneously on 6 dates from June–August, 2016. For vegetation sampling, inflorescences were counted for each species of wildflower occurring within a randomly selected 0.25 m^2^ quadrat within each subplot. Arthropod counts were carried out within the same quadrat by visually observing flower visits for 3 min. Observations were classified into one of three functional groups of arthropods: pollinators, natural enemies and herbivores. All bees and syrphid flies visiting the flowers were grouped as pollinators, spiders and insect predators (Reduviidae, Geocoridae, Carabidae, Coccinellidae, Dermaptera and Hymenopteran parasitoid wasps) were grouped together as natural enemies. Most of the Hemiptera (other than the known predatory taxa like Geocoridae, Reduviidae) and all orthopterans were counted as herbivores. Of the 20 buffer plots, one failed to establish due to a combination of excess soil water and continual disturbance from farm equipment traffic in adjacent fields. Consequently, we present results of the four response variables (counts of wildflowers, pollinators, natural enemies, and herbivores) from 19 plots where data collection was feasible.

### 2.3. Bioenergy Production and Soil Nutrients

Napier grass was harvested and weighed in-field with an adapted tractor containing cutting blades, a conveyor shaft, chopper, and a weighing scale following senescence (13 January 2017). Subsamples of the cut grass were collected and dried to obtain dry weights and buffer site specific yields (kg/ha) were estimated based on the size of the area sampled. Soil samples were collected from all plots on 23 June 2016. For each plot, ten soil cores were extracted from a depth of 15 cm at pre-selected randomized locations and homogenized to form one sample per plot. Samples were sent to the UGA Agricultural and Environmental Services Laboratories for processing and testing for nutrients (LBC, K, Ca, Mg, Zn, Mn, P) and pH. Lime buffer capacity (LBC) is a measure of the amount of soil acidity that must be neutralized in ppm by pure calcium carbonate to raise the pH by one unit.

### 2.4. Statistical Analyses

Spatial autocorrelation was assessed with Mantel’s test using “ade4” in R for each arthropod response variable (i.e., pollinators, natural enemies and herbivores). Generalized Least Squares (GLS) were fit to square root transformed response variables (i.e., wildflowers, pollinators, natural enemies and herbivores) using “gls{nlme}” within the statistical software R [25,26]. This model setting allowed for unequal variance structure and provided best spread of residuals and stable parameter estimates. Random effects were specified as sample data nested in sample plots to account for repeated measures of plots over time, which was best modeled with correlation structure of errors using corAR1{nlme}. Fixed-effect predictor variables were specified as the experimental design: local spatial context treatment (i.e., buffer adjacent to agriculture or woodland), and irrigation treatment (irrigated or non-irrigated), wildflower treatment (control, M1, M2, M3), sampling date, initially all two-way interactions and three-way interactions. Model reduction was accomplished by using Akaike Information Criterion, Bayesian Information Criterion (BIC), and log likelihood estimates from model fitting. The best fitting model was reported, and adjusted multiple comparisons for significant main effects were evaluated using “lsmeans” with the Tukey method.

Napier grass dry mass yields and total seasonal production of flowers (total number of flowers in the entire plot) were analyzed in relation to soil covariates and location by irrigation treatment design. Correlations between each of the soil covariates were assessed and variables with strong correlations were removed to eliminate multicollinearity. To standardize covariates, each was natural log transformed. In a second step, we fit a linear model containing the covariates (LBC, pH, K, Mg, Mn, and P), location treatment (agriculture or wooded margin) and irrigation treatment (irrigated or dryland), and ranked the inclusion of different combinations of covariates, treatments, and interactions using stepAIC (with forward and backward selection). The best fitting model was reported, and adjusted multiple comparisons for significant main effects were evaluated using “lsmeans” with the Tukey method.

## 3. Results

### 3.1. Wildflowers

**Figure 2:**
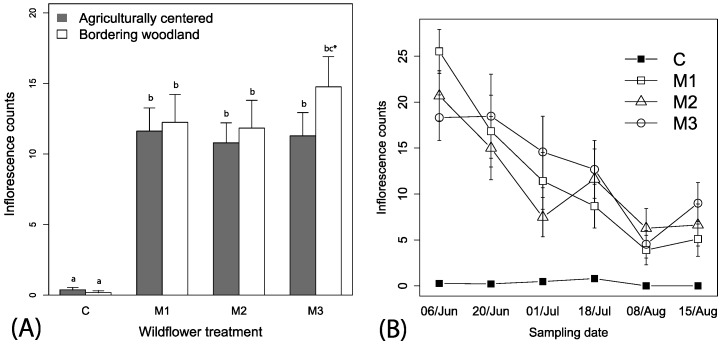
Mean (+1 SE) observations of (**A**) inflorescences among the mixes in relation to local spatial context, and (**B**) across time. Legend labels: C—control, M1—floral mix, M2—floral mix, and M3—floral mix; Table S1). Lowercase letters in panel (**A**) represent adjusted linear comparisons α = 0.05, where all wildflower treatments (M1, M2, M3) were significantly different from the control (**C**), and there was a tendency for there to be more flowers in plots adjacent to woodlands for M3 (indicated by bc *).

We observed nearly every flower species included in the commercial mixes, some with bloom periods extending over the entire season (Table S1, Table 1). *Coreopsis tinctoria* appeared to yield more flowers earlier in the season while *Gaillardia pulchella* and *Rudbeckia hirta* flowered throughout the season (Table 1). Best fitting model of wildflower counts contained the mix treatment, date and the interaction between the mix treatment and date (i.e., ΔAIC > 10 for other models), but we retained local context and irrigation for ease of reporting. Significantly more inflorescences were observed in the subplots with commercial mixes as compared to the control treatments containing weeds (*F*_3, 446_ = 39.23, *p* < 0.001, Figure 2A). Inflorescence counts were not significantly influenced by irrigation (*F*_1, 446_ = 0.12, *p* = 0.723), and there was a numerical tendency for higher numbers in wooded margins (*F*_1, 446_ = 2.25, *p* = 0.134, Figure 2A). An interaction between wildflower treatment and time showed decreasing numbers of flowers between wildflower mixes and no change in the control over time (*F*_3, 375_ = 12.33, *p* < 0.001, Figure 2B); slopes (coefficient estimate for mixes ranging between −0.305 (0.08) to −0.47 (08), *t*-value = −3.67 to −5.51, *p* < 0.001).

### 3.2. Pollinators

**Figure 3:**
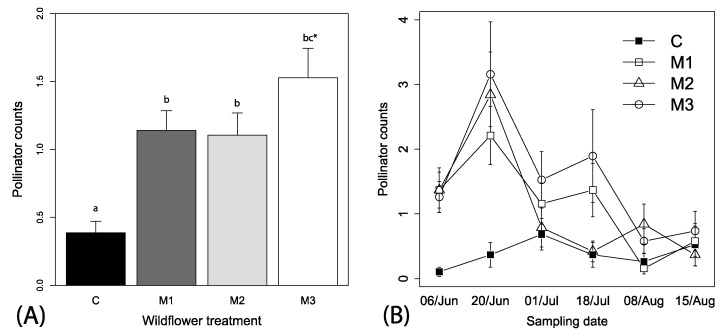
Mean (+1 SE) observations of (**A**) pollinators among the mixes and (**B**) mean (±1 SE) pollinators across the time among the different wildflower treatments. Wildflower treatments represent same as Figure 1; Table S1). Lowercase letters in panel (**A**) represent adjusted linear contrasts at α = 0.05.

Spatial autocorrelation was not found for pollinators in relation to site locations (Mantel’s test, r^2^ = −0.024, *p* = 0.558). A total of 474 pollinators (bees and syrphid flies) were visually observed over the season, which ranged from 0 to 13 individuals per observation. The best fitting models relating explanatory variables to pollinator counts included the variables wildflower mix, week and the interaction between mix and week (i.e., Δ AIC > 10 for other models). We retained irrigation and local context in the models because these did not impact parameter estimations and allowed for ease of presentation. Overall, significantly higher numbers of pollinators were observed visiting the wildflower treatment plots than control plots (*F*_3, 446_ = 16.32, *p* < 0.001, Figure 3A), independent of local context (*F*_1, 446_ = 0.1.61, *p* = 0.204) or irrigation treatments (*F*_1, 446_ = 0.03, *p* = 0.855). There was also a tendency for higher numbers of pollinators visiting the M3 than the M1 and M2 wildflower treatments (Figure 3A). A significant interaction between sampling date and wildflower treatments indicates a time dependency of effects of wildflower availability on pollinator visits, with an overall decreasing pattern of fewer pollinators visiting wildflower plots overtime (*F*_3, 446_= 8.15, *p* < 0.0001, Figure 3B; slope coefficient estimate = −0.10 (0.02), *t*-value = −3.49, *p* < 0.001).

### 3.3. Natural Enemies

**Figure 4:**
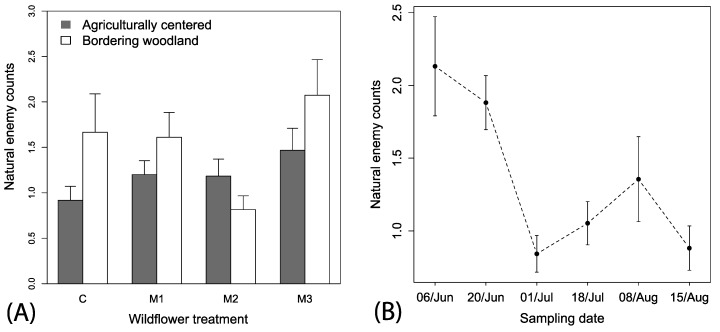
Mean (+1 SE) observations of (**A**) natural enemies among the mixes and local spatial context and (**B**) mean (±1 SE) natural enemies across time.

Predator counts were not spatially autocorrelated with site locations (Mantel’s test, r^2^ = 0.38, *p* = 0.314). A total of 619 natural enemies (Araneae, hymenopteran parasitoids, Coccinellidae, Geocoridae, and Nabidae) were observed in wildflower plots. Natural enemy counts were variable, but the best fitting model suggests populations were changing over time and there were interactive effects of local context and wildflower mix (i.e., ΔAIC > 3 for other models; Figure 4A,B). There was an overall tendency for natural enemies to be observed more frequently in plots with wooded borders (*F*_1, 446_ = 3.17, *p* < 0.076, Figure 4A) and an interaction between local context and wildflower mix included in the model was the best fitting, but not statistically significant (*F*_3, 446_ = 1.65, *p* = 0.177), which indicates a tendency for higher numbers of natural enemies in wooded bordered plots in M1, and M3, but lower than expected in M2 (Figure 4A). There was a strong significant seasonal time effect of decreasing and fluctuating numbers of natural enemies (*F*_1, 446_ = 20.44, *p* < 0.001, coefficient estimate = −0.06 (0.01), *t*-value = 4.52; Figure 4B).

### 3.4. Herbivores

**Figure 5:**
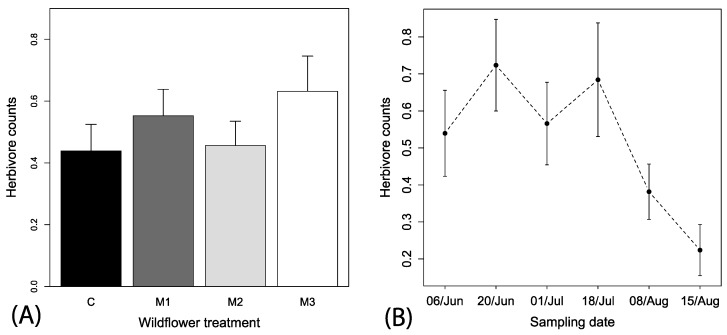
Mean (+1 SE) observations of (**A**) herbivores among the mixes and local spatial context and irrigation treatments and (**B**) mean (±1 SE) herbivores across the sampling periods among the different wildflower treatments. Local spatial context and irrigation descriptions are the same as in Figure 2.

Spatial autocorrelation was also not observed for herbivore observations (Mantel’s test, r^2^ = 0.019, *p* = 0.358). A total of 237 hemipteran herbivores were observed on flowers over the season. The best fitting model explaining herbivore counts included only the predictor variable week (i.e., ΔAIC > 3 for other models), but there was a tendency for higher numbers of herbivores in M3 plots. Consequently, we retained all explanatory variables in the model and removed all interactions that reduced model adequacy to allow for ease of presentation. Spatial context (*F*_1, 446_ = 0.35, *p* < 0.553) or irrigation treatments (*F*_1, 446_ = 0.04, *p* < 0.836) had no significant effect on herbivore counts. Wildflower treatment was included in better fitting models that were closer to the best fitting and indicate a tendency for higher numbers of herbivores in the M3 subplot as compared to control (*F*_1, 446_ = 1.80, *p* < 0.145; Figure 5A). Time was a strong predictor of the counts of herbivores (*F*_1, 446_ = 14.70, *p* < 0.001) and a negative slope indicated an overall decreasing number of herbivores observed over the season (coefficient estimate = −0.3 (0.01), *t*-value = −3.75, *p* < 0.001).

### 3.5. Bioenergy Production, Widlflower Production and Soil Nutrients

**Figure 6:**
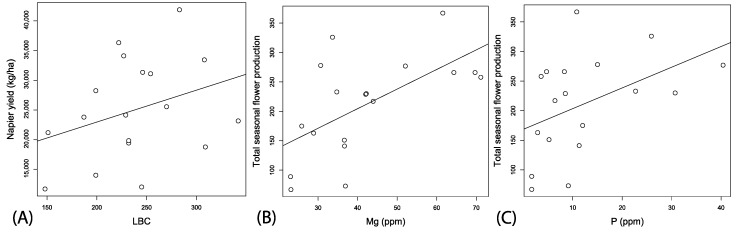
Correlation between estimated Napier yields from field buffer plots and LBC (**A**). Correlations between soil nutrients and total seasonal production of wildflowers pooled for the entire plot in relation to Mg (**B**), and P (**C**).

In this first year of planting Napier grass into the field locations, lime buffer capacity (LBC) was the only soil parameter serving as a significant predictor variable for yield (Table 2; ΔAIC > 2; *F*_1, 17_ = 4.11, *p* = 0.058, Adj. r^2^ = 0.15, Figure 6A). Local spatial context had no significant effect on Napier yields (Table 2). However, the best fitting model for predicting wildflower production contained the predictor variables: pH, Mg, and *p* (*F*_1, 13_ = 6.41, *p* = 0.004, Adj. r^2^ = 0.56). Total wildflower counts were non-significantly correlated with pH, coefficient est. = −3.20 (2.21), *t*-value = −1.45, *p* = 0.1697, significantly positively correlated with Mg, coefficient est. = 5.53 (1.41), *t*-value = 3.91, *p* = 0.002, and P, coefficient est. = 4.27 (1.42), *t*-value = 2.99, *p* = 0.010 (Figure 6B,C).

## 4. Discussion

Applied research has discovered agricultural benefits of habitat management through planting of native grasses and wildflowers, resulting in a demand for plant material. In response, seed companies are introducing annual and perennial seed wildflower mixes. Wildflower mixes are designed to provide regionally specific native flowering plants that help support and enhance native pollinator populations. Here, we tested three seed mixes containing similar combinations of southeast annual and perennial species in contrasting landscape contexts. We found each performed well in establishing and providing much higher numbers of flowers than spontaneous weeds. Although landscape context did not significantly influence the number of flowers produced, soil nutrient levels were an important predictor of overall wildflower production. In addition, as a second component of a landscape design concept, we established a perennial bioenergy grass, Napier grass, and, while the first year of yield data is insufficient for ascertaining its production potential, we suggest this conceptual design could enhance multiple ecosystem services in agricultural landscapes. Further longitudinal research manipulating the design parameters of such systems will better quantify costs and benefits associated with biofeedstocks-wildflower buffer plantings.

The increase in flowers produced in wildflower plots corresponded directly with increases in the number of pollinators observed in plots, and provide similar enhancement to pollinator numbers independent of location to agriculture or a forested border. Conversely, natural enemies were variable in response to wildflower plots, but there was a tendency for higher numbers in plots associated with a wooded margin. Herbivore insect numbers were slightly higher on one mix that tended to produce more flowers, and similar to other functional groups, fewer were observed later in the season. Our results suggest improving pollinator numbers is dependent upon providing more flowers over the season, but improving natural enemy populations for conservation biological control programs is not as straightforward.

Although there was an overall tendency for natural enemies to be observed more frequently in plots with wooded borders, location context had no effect on pollinator, natural enemy, and herbivore attraction to flowers. This is contrary to what has been found in other studies for pollinators and natural enemies [10,25]. However, this could be due to a lag period in responses, as maximum effects of floral provisioning on pollinators can take multiple years following establishment (e.g., [9,10]), which may be due to changes in the arthropod community composition or plant communities during establishment. Because of this lag effect, the response of pollinators, natural enemies, and herbivores to location context will likely change over time. Additionally, as pollinator and natural enemy populations are influenced by landscape context, broader landscape ecological composition may influence overall trends in local scale responses of arthropod species, with lower group diversity found in landscapes with less heterogeneity [27,28]. Thus, information about surrounding land cover and land use would be needed to predict responses of the taxa in different landscapes.

We also found no effect of irrigation on pollinator, natural enemy, and herbivore attraction to flowers, or Napier grass yield. Precipitation level over the course of our study was highly variable (mean ± SD of 3.33 ± 12.39 mm/day), but intervals of precipitation never exceeded one week (http://www.georgiaweather.net/, and Bosch, unpublished data). Therefore, there may have been sufficient precipitation for the plants in the non-irrigated plots during the season of our study.

The wildflower mixes in our study were dominated by three species in the Asteracae family: *Coreopsis tinctoria*, *Gaillardia pulchella* and *Rudbeckia hirta*, which are highly attractive to bumble bees (e.g., [29,30]). *Gaillardia pulchella* and *R. hirta* bloomed through the entire season and *C. tinctoria* bloomed early in the season, showing flower resources were present throughout the season. Further study is needed to determine whether dominant flowering plant nectar is accessible and has suitable nutrition for pollinators and natural enemies [31]. Increasing flower presence with extrafloral nectaries in flower strips may be one method to improve overall accessibility to nectar resources [5].

Pollinators were more abundant early in the season (mid-June) in all three of the native wildflower commercial mixes. Several crops in the region, flower early in the season and rely on pollinators (e.g., blueberry and watermelon) or produce higher quality fruit when pollinated (e.g., cotton). The early blooming pattern of high value crops coinciding with higher numbers of pollinators underscores the value of maintaining habitat for pollinators near these crops for potential improvements in crop production. Pollinator richness and visitation rates drastically decline to less than 50% of maximum value within fields at around ~1.5 km and ~600 m respectively from natural habitats [32]. Therefore, placement of floral resources must be carried out in such a way that spatial connectivity is taken into consideration.

Natural enemy numbers in the floral mixes tended to be more variable and not as clearly influenced by wildflower treatments as compared to pollinator numbers. However, the overall seasonal pattern for natural enemies was decreasing over the season, which was similar to bloom period of the wildflowers. Many predators are omnivores, and along with parasitoids, have competing needs for resources such as refuges, mating sites, hosts and alternative food resources. Natural enemies may be responding to a variety of cues from these areas, whereas pollinators are likely responding more to visual and chemical cues associated with flowers. Frank et al. [33] also found variable abundances of natural enemies over time, and showed natural enemy abundance does not always coincide with floral bloom periods. These results indicate some plant species can provide season long benefit by attracting natural enemies even if they are not blooming and emphasizes the need to understand natural enemy-plant associations that may enhance pest control (e.g., [34]).

We did not detect any definitive pattern of herbivores across the location context, irrigation, and wildflower treatments, and their mean counts were low. Encouragingly, pestiferous herbivores are expected to prefer crops to wildflower habitats [35]. It may also be that natural enemies were suppressing herbivore populations in our wildflower and control plots, but this will need further study. Our visual observations likely underestimated arthropod abundance in the wildflower habitat and in particular the herbivores that were only observed on flowers. Fielder and Landis [36] found numerous natural enemies and herbivores on native flowers by using plant suction sampling.

Lastly, in this first year of establishment, the site-specific yields of Napier grass were high and typical of yield ranges found for this species [37]. Napier grass yields were positively associated with LBC, the soil buffering capacity. We suspected pH would influence plot establishment because one of our sites had visibly grey soils and very weak vegetative growth, and the pH was 4.5; applications of lime to this plot greatly increased Napier grass and wildflower growth suggesting in some cases, lime amendment to soils may be needed for successful establishment. Overall, very little input was needed for Napier grass establishment and wildflower production in the first season of this project. Combining the Napier grass and wildflower habitat indicated the potential for simultaneously obtaining high biomass for biofuel production and habitat attractive to pollinators. Longitudinal data and higher resolution data on natural enemy and herbivore numbers is needed to more fully understand their response to these buffers over time.

## 5. Conclusions and Future Research

Over the first year of buffer establishment we saw substantial pollinator attraction to native wildflower treatments, but natural enemy and herbivore attraction to wildflowers was no different from control plots with spontaneous weeds. Wildflower and Napier grass establishment was high over all 19 sites and Napier grass yields were also high, which is common for in first-year establishment of Napier grass (W.F. Anderson, personal communication). Although we concentrated our study at the local scale, future studies will examine landscape effects on arthropod community patterns. Further study is also needed to examine the effects of buffer size and configuration on arthropod responses, and to quantify natural enemy pest control of annual crops. Interestingly, we found nutrients influence both yield and wildflower production, and suggests site characteristics should be assessed prior to establishment and some investment in pH and nutrient level adjustments may be needed to maximize production of biomass and flowers. Further analysis of the energy, resource, and benefit tradeoffs is required.

## Figures and Tables

**Table 1:** The bloom period and the total counts of inflorescences observed. The symbols indicate the presence of the respective floral species in one of the planted mixtures (□—M1; ∆—M 2; ○—M3).

Wildflower Species	Presence in Mix	6-Jun	20-Jun	1-Jul	18-Jul	8-Aug	15-Aug	Total Flowers
*Cynoglossum amabile*	∆○	8	2					10
*Eschscholzia californica*	□∆○		3					3
*Achillea millefolium*	□∆○		6					6
*Salvia coccinea*	□∆○		4				6	10
*Cosmos bipinnatus*	□∆○			7				7
*Echinacea purpurea*	□∆○			3				3
*Nemophilia maculata*	□			2				2
*Coreopsis tinctoria*	□∆○	490	289	210	4			993
*Phlox drummondi*	□∆○	57		12				69
*Rudbeckia amplexicaulis*	∆○		29	13				42
*Linum grandiflorum r.*	□∆○		9		1	13		23
*Centaurea cyanus*	□	91	25	27	16			159
*Rudbeckia gloriosa*	□∆○		3	12	7	6		28
*Oenothera lamarckiana*	∆○		5	6	3	5		19
*Cosmos sulphureus*	□	8	15	21	15	2	4	101
*Monarda citriodora*	□	75	61	12	11	4		163
*Coreopsis lanceolata*	□∆○	63	93	19	32	12	2	221
*Gaillardia pulchella*	□∆○	161	177	103	405	218	325	1389
*Rudbeckia hirta*	□∆○	278	239	198	148	19	21	903
Total flowers		1231	960	645	642	279	394	4151

**Table 2:** Mean (±1 SE) Napier grass yields and soil nutrient levels observed in plots in relation to local spatial context. Notes: adjacent to woodland (T) or agriculture (A); irrigated (I) and not irrigated (N). Lime buffer capacity (LBC) is a measure of the amount of soil acidity that must be neutralized in ppm by pure calcium carbonate to raise the pH by one unit.

Local Spatial Context Treatments	Napier Yield kg/ha			Mehlich 1 mg/kg (ppm)					
		LBC	pH	Ca	K	Mg	Mn	P	Zn
AI	27,462 (4527)	278 (22)	4.87 (0.09)	339 (37)	53.66 (10.23)	55.29 (6.30)	8.08 (2.19)	7.86 (1.06)	4.93 (2.05)
AN	22,590 (4797)	211 (18)	4.71 (0.09)	205 (21)	34.96 (8.21)	33.08 (3.27)	4.34 (0.92)	9.42 (3.76)	1.48 (0.31)
TI	26,661 (2641)	211 (33)	5.07 (0.20)	330 (61)	30.46 (8.78)	45.16 (8.98)	7.37 (2.53)	13.63 (6.22)	2.31 (0.93)
TN	25,688 (3966)	271 (25)	4.74 (0.16)	487 (202)	32.93 (6.55)	38.28 (6.83)	7.61 (1.80)	90.78 (71.08)	4.97 (1.96)

## Supplementary Materials

The following are available online at www.mdpi.com/2075-4450/8/4/104/s1.
