# Supplementary material for: Perennial Grass and Native Wildflowers: A Synergistic Approach to Habitat Management
Source: Insects. 2017 Sep 22;8(4):104. doi: 10.3390/insects8040104 (PMC5746787; doi:10.3390/insects8040104)
Supplement: Supplementary file 1 [file insects-08-00104-s001.pdf]

# Supplementary Materials: Perennial Grass and Native Wildflowers: A Synergistic Approach to Habitat Management

**Shereen S. Xavier, Dawn M. Olson, Alisa W. Coffin, Timothy C. Strickland and Jason M. Schmidt**

**Table S1.** Wildflower species in the different commercial mixtures.

| wildflower species | M1 | M2 | M3 | Type |
|---|---|---|---|---|
| *Achillea millefolium* | × | × | × | Perennial |
| *Cosmos bipinnatus* | × | × | × | Annual |
| *Centaurea cyanus* | × | | | Annual |
| *Cheiranthus allionii* | × | × | × | biennial |
| *Chrysanthemum maximum* | | × | × | Perennial |
| *Coreopsis lanceolata* | × | × | × | Perennial |
| *Coreopsis tinctoria* | × | × | × | Annual |
| *Cosmos sulphureus* | × | | × | Annual |
| *Cynoglossum amabile* | | × | × | Perennial |
| *Dianthus barbatus* | | × | × | Perennial |
| *Dimorphotheca sinuata* | × | | | Annual |
| *Echinacea purpurea* | × | × | × | Perennial |
| *Eschscholzia californica* | × | × | × | Perennial |
| *Gaillardia aristata* | × | | | Perennial |
| *Gaillardia pulchella* | × | × | × | Annual |
| *Gilia capitata* | | × | × | Annual |
| *Lavatera trimestris* | | × | × | Annual |
| *Liatris spicata* | × | × | × | Perennial |
| *Linaria maroccana* | × | | | Annual |
| *Linum grandiflorum rubrum* | × | × | × | Annual |
| *Linum perenne lewisii* | | × | × | Perennial |
| *Linum usitatissimum* | × | | | Annual |
| *Lobularia maritima* | × | × | × | Perennial |
| *Lupinus perennis* | × | × | × | Perennial |
| *Lupinus polyphyllus* | × | | | Perennial |
| *Lupinus texensis* | | × | × | Annual |
| *Monarda citriodora* | × | | | Annual |
| *Nemophila maculata* | × | | | Annual |
| *Oenothera lamarckiana* | | × | × | Annual |
| *Papaver rhoeas* | × | × | × | Annual |
| *Phlox drummondii* | × | × | × | Annual |
| *Rudbeckia amplexicaulis* | | × | × | Annual |
| *Rudbeckia gloriosa* | × | × | × | Perennial |
| *Rudbeckia hirta* | × | × | × | Annual |
| *Salvia coccinea* | × | × | × | Perennial |
